# Assessment of Air Pollutant Concentrations Near Major Roads in Residential, Commercial and Industrial Areas in Ibadan City, Nigeria

**DOI:** 10.5696/2156-9614-7-13.11

**Published:** 2017-03-29

**Authors:** Ayodele Rotimi Ipeaiyeda, Dayo Amos Adegboyega

**Affiliations:** Department of Chemistry, University of Ibadan, Ibadan, Nigeria

**Keywords:** sulphur dioxide, NOx, ozone, ammonia, carbon monoxide, industrial and non-industrial areas

## Abstract

**Background.:**

Urbanization, energy consumption, intensification of road traffic and rapid population growth pose significant challenges to ambient air quality. Therefore, regular monitoring of current air quality is essential.

**Objectives.:**

The study investigated the concentration of sulfur dioxide (SO_2_), oxides of nitrogen (NOx), ozone (O_3_), ammonia (NH_3_) and carbon monoxide (CO) in ten different sites to investigate possible anthropogenic sources within Ibadan, Nigeria.

**Methods.:**

Air samples were collected into specific absorbing solutions twice daily in the morning and afternoon for four weeks. The resulting solutions were analyzed following specific colorimetric techniques according to standard methods of analysis.

**Results.:**

Average concentrations varied among the sampling areas. All were above background levels obtained at the control site. Overall concentrations were found to be 30±19 μg/m^3^ (SO_2_), 63±16 μg/m^3^ (NOx), 31±18 μg/m^3^ (O_3_), 463±180 μg/m^3^ (NH_3_) and 0.59±0.21 ppm (CO). These concentrations were present at the study areas at 15, 31, 17, 5 and 3 times the background concentrations for SO_2_, NOx, O_3_, NH_3_ and CO, respectively.

**Conclusions.:**

In spite of the short duration of sampling, the data can guide future long term monitoring of air quality in Nigeria. The level of SO_2_ exceeded World Health Organization limits within this short duration. This is an indication of the need for long term air quality monitoring with a sustainable plan for air pollution management.

## Introduction

Rapid population growth in cities, industrialization, development and intensification of road traffic pose significant challenges to ambient air quality.^1^ Poor urban air quality are traceable to toxic criteria air pollutants according to the US Clean Air Act of 1970.^2^ Six air pollutants which are designated as criteria pollutants are sulphur dioxide (SO_2_), nitrogen dioxide (NO_2_), carbon monoxide (CO), ozone (O_3_), particulate matter with aerodynamic diameters under 10 and 2.5 μm, as well as lead. These are regarded as criteria pollutants because they are strongly suspected to be harmful to public health and the environment. There are one hundred and eighty-nine other potentially harmful pollutants that are designated as toxic or hazardous air pollutants. These pollutants are prevalent in the environment and can be discharged by sudden accidental releases. Some hazardous air pollutants fall into the category of volatile organic compounds.^3^

Sources of air pollutant emissions differ from one pollutant to another. Sulphur dioxide is emitted into the atmosphere in large quantities by chemical industries, vehicular traffic and petroleum refining. Vehicular emissions were identified as one of the major sources of SO_2_ and NO_2_, contributing to air pollution in the Jharia coal field in Jharkhand, India.^4^ Oxides of nitrogen (NOx) are formed when fossil fuels are burned at high temperatures. Much of the NOx in urban areas comes from vehicular emissions. Notable levels of ozone and NOx in the ambient air around highways in the US East Coast has been observed.^5^

Agriculture is the dominant source of ammonia emissions. Ammonia (NH_3_) is usually released from animal waste and fertilizer use. Carbon monoxide emission is usually due to incomplete burning of carbon-based fuels, including petrol, oil and wood. It is also produced from synthetic products such as cigarette smoke. High levels of CO can be noticed in poorly ventilated residential areas and along roadsides in heavy traffic.^6^ The occurrence of CO in residential homes and commercial sites in Delhi, India results principally from cooking using biomass as fuels and manual cleaning of homes.^7^ Ozone, the only secondary pollutant monitored in this study, is formed by chemical reactions between NOx and volatile organic compounds in the presence of sunlight. These air pollutants were selected for this study because of the mounting evidence of their involvement in exacerbating health challenges.^8^

The health effects of air pollution from fossil fuel combustion have long been known. Long term exposure to air pollutants causes respiratory and cardiovascular diseases.^9^,^10^ The risk of respiratory illnesses such as allergies, asthma, chronic obstructive pulmonary disease and lung cancer increases with exposure to atmospheric air pollutants.^11^,^12^ Economic development, urbanization, energy consumption, transportation and rapid population growth have been identified as anthropogenic activities contributing to air pollution.^13^ Research has shown that children and the elderly are particularly vulnerable to the health effects of air pollutants such as O_3_, particulate matter and other airborne toxicants.^10^,^14^,^15^ Similarly, exposure to high concentrations of NOx and CO is associated with adverse impacts. The control of airborne toxicants, particularly CO and NOx concentrations, is a major concern in some developed countries.^16^ Air pollution levels in some other developed countries have been decreasing dramatically in recent decades. Many cities in developing countries such as Nigeria have been suffering from increasing air pollution since the discovery of oil. Routine gas flaring continues and the practice gives rise to atmospheric contamination. In addition, there is high rate of rural to urban migration, which results in problems like traffic congestion, squatter settlements, as well as poor air quality. Ibadan, the second largest city in Nigeria in terms of landmass, has experienced such an increase in population growth.^17^ In 1951, the population of Ibadan was 100,000, with buildings occupying an area of about 35 square kilometers. The population rose to about 2,550,593 in 2006.^18^,^19^ Sulphur dioxide levels have been reported for Ibadan city in 2001 and 2013.^20^,^21^ Since then, there have been no further reports on levels of SO_2_ and other air pollutant emissions for Ibadan city. In addition, studies have been limited in scope and duration in terms of sampling.

**Abbreviations**
*CO*: Carbon monoxide
*NH*_*3*_: Ammonia
*NO*: Nitric oxide
*NO*_*2*_: Nitrogen dioxide
*NOx*: Oxides of nitrogen
*O*_*3*_: Ozone
*SO*_*2*_: Sulfur dioxide
*UI*: University of Ibadan

The vehicle population of Ibadan has increased at an alarming rate with a very high number of cabs. The principal industries associated with air pollution in the city concentrated in the industrial area include food and beverages, paint, and pharmaceuticals. Regulatory control strategies and policies are required to considerably reduce air pollution around the world. These controls must be based on an understanding of pollution characteristics and airborne toxicants from various pollution sources. However, there is inadequate air quality monitoring in many megacities such as Ibadan. Obvious possible sources of air pollution in Ibadan include motor vehicles, industrial processes, domestic heating and coal burning in commercial areas. Studies have indicated that vehicles are the major source of air pollution in most cities.^4^,^22^,^23^ The major reasons for the high level of vehicular emission are the large number of vehicles on congested streets, poor quality vehicles, poor quality fuels and maintenance systems and ineffective transport management. These reasons are evident in Ibadan city, although it is possible that the intensity of vehicle congestion, manufacturing industries and domestic heating varies across the different residential, commercial and industrial areas. There may be daily variations in air pollutant emission patterns, especially in the vicinity of highways or roadsides.

The objective of this study was to assess the status of NOx, SO_2_, CO, O_3_, and NH_3_ near major roads in residential, commercial and industrial areas in Ibadan city. These air pollutants were selected for this study because of their association with source categories of domestic heating, traffic and industrial facilities. A number of case studies with similar objectives have been conducted in other parts of the world.^24–27^ Due to increased awareness of the health effects of air pollution exposure, it is important that the public understand the day-to-day air quality conditions to which they are exposed. This study will provide a database to assist the air regulatory body in improving policy making and weather forecasting as well as provide rationale for mitigation measures.

## Methods

### Sampling Locations

**Figure 1:**
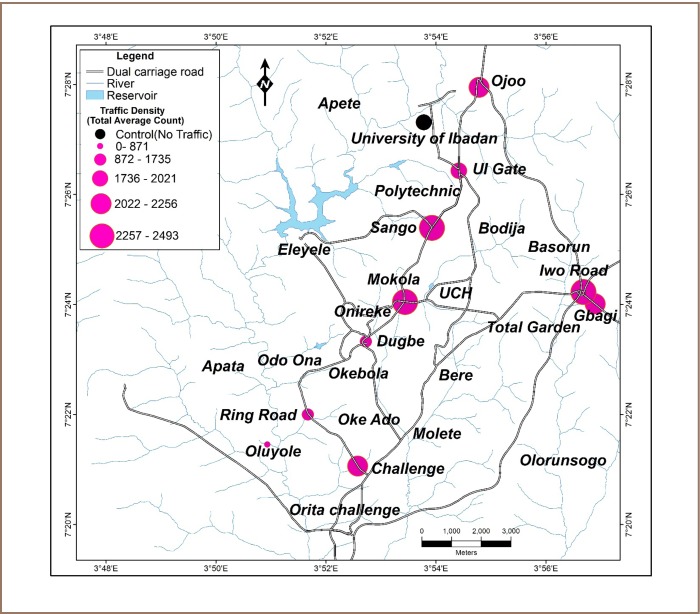
Map of Ibadan showing sampling areas near major roads

Ten sampling locations within Ibadan city (*Figure 1*) were purposively selected based on location features and human activities identified by the town planning authority, focusing on commercial, residential and industrial activities. The dual carriage roads shown in Figure 1 are surrounded by smaller main and secondary roads. A botanical garden located at the University of Ibadan was used as a control site. This site was far away from the major pollution sources under investigation. It is a reserved area with no trace of industry or residential areas and is not affected by extraneous local automobile emissions. The selection of other sampling locations and study design are in line with the objective of identifying anthropogenic sources of selected air pollutants and assessing their levels. All sampling locations were in major commercial, residential and industrial areas traversed by highways. Sampling locations are further described in Table 1.

**Table 1:** Mean Concentrations of SO_2_, NOx, O_3_, NH_3_ and CO with Traffic Density at Each Sampling Location

Sampling location	Location features	GPS (Latitude; Longitude)	Traffic density	SO_2_	NOx	O_3_	NH_3_	CO
	
				μg/m^3^				(ppm)
Ojoo	Commercial & Residential	N07^°^ 46^′^; E 003^°^ 91^′^	2153	20.0±8.0^bc^	50.1±0.2^c^	41±18^de^	527±190^c^	0.59±0.34^c^
UI Gate	Commercial	N07^°^ 39^′^; E003^°^ 90^′^	2021	26±10^cd^	65.6±0.7^de^	14.5±0.2^ab^	272±250^b^	0.75±0.02^c^
Sango	Commercial	N07^°^42^′^; E 003^°^ 89^′^	2419	49±12^e^	35.9±0.5^b^	20.8±4.7^cd^	649±28^cd^	0.65±0.15^c^
Mokola	Residential	N07^°^ 40^′^; E 003^°^ 89^′^	2271	50.8±2.0^e^	72.4±1.5^ef^	38±19^de^	689±59^d^	1.01±0.16^d^
Iwo Road	Commercial	N07^°^ 40^′^; E003^°^ 94^′^	2493	15.6±0.6^bc^	72.2±2.5^ef^	33±28^de^	331±150^b^	0.34±0.09^b^
Gbagi	Commercial	N07^°^ 39^′^; E 003^°^ 94/	2253	34±11^d^	51.3±0.5^c^	34.5±6.6^de^	329±62^b^	0.30±0.07^b^
Challenge	Commercial	N07^°^ 40^′^; E 003^°^ 95^′^	2197	17.6±1.1^bc^	81.7±1.3^f^	15.7±4.0^ab^	344±57^b^	0.63±0.21^c^
Ring Road	Commercial	N07^°^ 36/; E 003^°^.86^′^	1735	13.6±2.3^bc^	84.0±0.9^f^	2.06±0.73^a^	216±16^ab^	0.70±0.17^c^
Dugbe	Commercial	N07^°^ 39^′^; E 003^°^ 88^′^	1545	8.39±0.57^ab^	51.3±1.0^c^	61±12^f^	629±21^cd^	0.35±0.10^b^
Oluyole	Industrial & Residential	N07^°^ 35^′^; E 003^°^85^′^	871	63±14^f^	58.2±1.4^cd^	53.0±2.2^ef^	648±11^cd^	0.64±0.12^c^
Botanical Garden (Control site)		N07^°^45^′^; E 003^°^89^′^	0.00	2.00±0.57^a^	2.3±0.1^a^	1.80±0.36^a^	91.4±5.2^a^	0.18±0.04^a^

Mean values within each column with different superscripts (a,b,c,d,e,f) are significantly different (P=0.05); Traffic density=number automobile per hour

### Sampling and Chemical Analysis

Sampling of SO_2_, NOx, O_3_, NH_3_ and CO from air was carried out using the absorption train technique.^28^ The technique involves the absorption of pollutant gas from air into a reagent solution.

Sampling was conducted twice a day (once in the morning and once in the afternoon) for four weeks. Air samples were collected from 8:00 to 11:00 in the morning and 16:00 to 19:00 in the afternoon. The absorption train consists of various components. The components include a set of flow rate calibrated critical orifices and impingers containing absorbing solutions for SO_2_, NOx, ozone, NH_3_ and CO, separately. The absorbing solutions were sodium tetrachloromercurate for SO_2_, acidic potassium dichloro and a mixture of N-(1-naphthyl)-ethylene diaminedihydrochloride and sulphamic acid in glacial acetic acid for NOx, dilute sulphuric acid for NH_3_, non-indicating silica gel impregnated with ammonium molybdate for CO and buffered potassium iodide for ozone. Air was bubbled with the air pump through an impinger containing the absorbing solution designed to react with each air contaminant. Each contaminant was collected in this way and appropriate laboratory analysis of the resulting solutions followed using standard methods.^29^,^30^ The resulting solutions were analyzed following the colorimetric technique. The technique involves using the modified West-Gaeke method for SO_2_, Griess-Saltzman method for NOx, Nesslerization method for NH_3_, neutral and alkali-iodide method for ozone and reduction of silico-molybdate method for CO.^31–35^

### Statistical Analysis of Analytical Data

Descriptive analysis was used for the analytical data. The Duncan multiple range test (α=0.05) was used to determine if the air pollutant level for one location was significantly different from another location. One-way analysis of variance (ANOVA) was used to determine the level of significance of the difference between the concentrations of pollutants in the morning and afternoon. A biplot diagram was adopted to illustrate the underlying relationship of pollutant concentrations with sampling location features. The aim is to represent the sampling locations and data of pollutant types in at least two dimensions so that a single multivariate visual impression may be obtained, with the calibrated biplot axes incorporating the original variables. The sampling locations are represented as points, while the concentrations of pollutant types are represented as labeled variables. The biplot uses vectors to represent the coefficients of the variables displayed in the plot. The pollution types that point in the same direction correspond to pollution types that have similar profiles in terms of source emission, and can be interpreted as having similar sources in the context set by the data. The first two principal component values explain the major part of the variance along the axes.

## Results

### Concentrations of Air Pollutants in Sampling Locations

Average NOx concentrations for all sampling locations ranged from 35.9±0.5 μg/m^3^ obtained at Sango to 84.0±0.9 μg/m^3^ obtained at Ring Road. The Sango sampling location had the lowest NOx concentration and is a residential area with few commercial activities and a traffic density of about 2419 vehicles/hr. The traffic density at Ring Road, a commercial area with the highest NOx concentration was 1735 vehicles/hr (*Table 1*). The background NOx concentration of 2.3±0.1 μg/m^3^ measured at the control site was low and differed significantly from corresponding concentrations obtained for all sampling locations (p>0.05). The overall average NOx concentration of 63±16 μg/m^3^ obtained in Ibadan is similar to NOx concentrations in most urban outdoor air, which have been found to fall within the range of 20–90 μg/m^3^.^36–38^

In this study, the maximum ozone concentration of 61±12 μg/m^3^ was observed at the Dugbe sampling location. This location is a commercial area with a traffic density of 1545 vehicles/hr. Elevated ozone concentrations of 33±28 μg/m^3^; 34.5±6.6 μg/m^3^ and 41±18 μg/m^3^ were also observed in other sampling locations with similar commercial activities (*Table 1*).

The only natural alkaline air pollutant is ammonia and it influences the pH of cloud water and rainfall. The NH_3_ emission from the residential area at Mokola was estimated to be 689±59 μg/m^3^. The emission from the residential area was not significantly different from the ammonia levels found at commercial and industrial areas (p>0.05). Ammonia emissions at the commercial area of Dugbe and Oluyele industrial estate were 629±21 μg/m^3^ and 648±11 μg/m^3^, respectively (*Table 1*). Background emission of ammonia at the control site was 91.4±5.2 μg/m^3^, which was considerably lower than NH_3_ emission levels at other investigated roadsides. An ammonia concentration of 463±180 μg/m^3^ was observed for Ibadan city (*Table 2, Table 3*).

**Table 2:** Comparison of Air Pollutant Levels Along Major Roads in Ibadan City with National Air Quality Guidelines

	SO_2_	NOx	O_3_	NH_3_	CO	Reference number
		
			μg/m^3^		ppm	
Ibadan, Nigeria	30±19	63±16	31±18	463±180	0.59±0.21	This study
California AAQS	655	339	180	-	20	57
India AAQS	80	80	180	-	-	49
WHO AQG	*20	200	-	-	-	50
NAAQS (USEPA)	196	188	-	-	35	57

Abbreviations: AAQS, Ambient Air Quality Standards; WHO AQG, World Health Organization Air Quality Guidelines; NAAQS, National Ambient Air Quality Standards; USEPA, United States Environmental Protection Agency.

**Table 3:** Comparison Air Pollutant Levels Along Major Roads in Ibadan, Nigeria with Results from Other Nigerian Locations and Other Countries

Sampling location	SO_2_	NOx	O_3_	NH_3_	CO	Reference number
		
	μg/m^3^				ppm	
Ibadan, Nigeria	30±19	63± 16	31±18	463±180	0.59±0.21	This study
Ibadan, Nigeria	34.1	-	-	-	-	20
Ibadan, SW, Nigeria	<0.262–1570.6	<0.188–564.4	-	-	<0.115–1145.2	21
Ilorin, Nigeria	155.22±4.97	-	-	-	-	51
Ilorin, Nigeria	11.07±1.36					52
Kaduna, Nigeria	110	89.0	-	-	14640	53
Port Harcourt, Nigeria	25	141.1			5725	54
NAFCON, Nigeria				33.6		55
Incheon, South Korea	17	71.4			566	41
Uttarakhand, India	15	23	-	-	-	56
Bursa, Turkey		78.8±70.4	55.1±32.5			38

Carbon monoxide concentrations resulting from vehicular emissions at sampling locations were in the range of 0.30±0.07 ppm to 1.01±0.16 ppm. The lowest average CO level was obtained at Gbagi with a traffic density of 2253 vehicles/hr, while there was a reported traffic density of 2271 vehicles/hr at Mokola where the highest CO level was obtained. Carbon monoxide emissions at all sampling locations were significantly higher than the background carbon monoxide level at the control site (*Table 1*). The overall CO emission level of 0.59±0.21 ppm was much lower than the United States Environmental Protection Agency guideline of 35 ppm (*Table 2*). The level of CO obtained along the roadside in the industrial area was 0.64±0.12 ppm. This was not significantly different from the corresponding CO levels obtained at Ring Road, Challenge, Sango, University of Ibadan (UI) gate and Ojoo, which were all commercial areas. The traffic density in the industrial area was 871 vehicles/hr, which was much lower than in the other sampling locations.

### Daily Variation of Pollutant Levels in Sampling Locations

**Figure 2:**
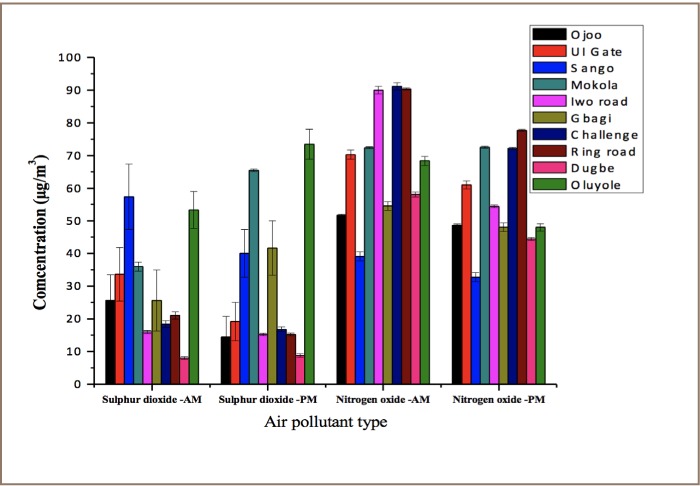
Daily variations of sulphur dioxide (SO_2_) and nitrogen oxide (NOx) concentrations

**Figure 3:**
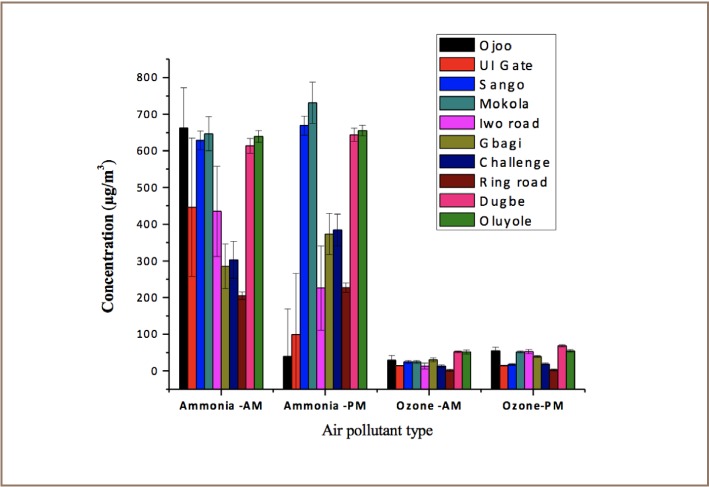
Daily variations of ammonia (NH_3_) and ozone (O_3_) concentrations

NOx concentrations observed in the afternoon were not significantly different (p>0.05) from the corresponding concentrations in the morning as shown in Figure 2. This observation differs with a report that found that low NOx concentrations were obtained in the afternoon.^38^ Figure 3 revealed that ozone formation was higher in the morning than in the afternoon. An increase in fossil fuel consumption by vehicles in the morning due to increased traffic could have led to an increase in ozone concentration. NOx and O_3_ are not strongly correlated (r = 0.0906), suggesting that NOx concentration did not increase at the desired quantity in all study areas to sufficiently support the required ozone formation. An ambient temperature range of 28.2–31.5^°^ C was observed during sampling (*Table 4*). Ozone concentrations in the morning were also observed to be generally low compared with the corresponding concentrations in the afternoon when the temperature values were high and relative humidity values (61–87%) were low.

**Table 4:** Mean Values of Meteorological Data Obtained Within the Sampling Period for Ibadan, Nigeria

Parameter	Mean (Range)
Average high temperature (°C)	31.9 (28.2 – 35.1)
Average low temperature (°C)	22.8 (21.3 – 23.7)
Daily sunshine hours (hours)	5.6 (2.8 – 6.9)
Mean daily evaporation (millimeters/day)	3.6 (1.7 – 7.3)
Mean wind speed (m/s) (meters/second)	1.32 (0.87 – 1.54)
Humidity (%)	79 (61 – 87)
Average precipitation per day (millimeters)	8.0 (2.0 – 12.0)

*Source*: NIMET Centre, Ibadan^58^

**Figure 4:**
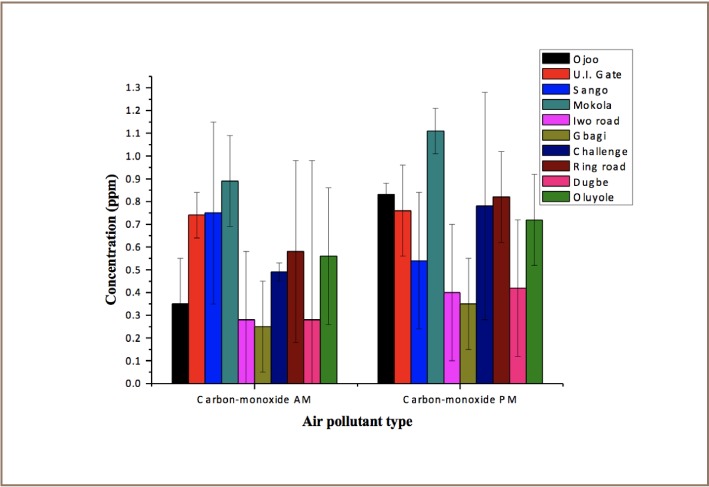
Daily variations of carbon monoxide concentrations

A high level of ammonia was observed during morning rush hours in a few locations such as Ojo, UI gate, Iwo road, Challenge and Ring road (*Figure 3*). There was a high level of ammonia in the afternoon compared to the morning for locations such as the Oluyole industrial area, Dugbe and Challenge. There was no significant difference in CO emissions in the morning and afternoon as revealed in Figure 2. Figure 4 reveals the considerable variability in atmospheric CO levels that exists in the day time. In this study, CO showed little change in the time of daily occurrence of its morning maxima except for the Sango location.

### Variation of Pollutant Levels for Different Sampling Locations

**Figure 5:**
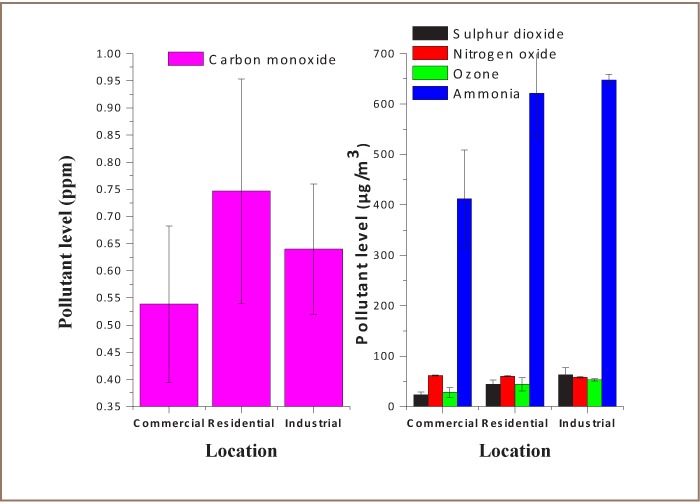
Variations of pollutants levels for different sampling locations

Small quantities of CO from natural sources have been identified in the past.^39^ The increase in atmospheric CO level has been caused by anthropogenic sources. Figure 5 shows that about 38.8%, 33.2% and 28.0% of the total CO emitted was associated with residential, industrial and commercial land usage based on this study. The second highest average traffic flow of 1770 vehicles/hr was observed for residential areas where buildings on both sides are closely packed. Emissions from homes and vehicles could be trapped within the residential areas such that dispersion of NOx and CO is restricted. The distributions of pollutant levels among the other source categories considered in this study are also shown in Figure 5. The proportions of SO_2_ variability were 17.6%, 34.2% and 48.2% for commercial, residential and industrial areas, respectively. The corresponding proportions for ozone were 22.3%, 35.3%, and 42.5%, and 24.5%, 36.9% and 38.5% for ammonia according to commercial, residential and industrial area, respectively. An equal proportion of about 33% of NOx was observed for each source category (*Figure 5*).

## Discussion

### Comparison of Pollutant Levels with Air Quality Standards and other Results

All air pollutants measured in this study were present at low levels compared with the national ambient air quality standards (*Table 2*). Since air has no boundaries, it is conceivable that much of the accumulated atmospheric pollutants from the various sources may eventually migrate by atmospheric convection to a potential sink in the atmosphere of another region or continent. Therefore, knowledge of how comparable the concentration of a specific air pollutant is in a region with another region or continent is valuable. SO_2_, NOx and O_3_ concentrations were comparable with the reported values obtained in Bursa, Turkey and Incheon, South Korea (*Table 3*). Oluyole industrial estate was found to have high ozone concentration traceable to industrial heating. An ozone concentration range of 20–60 ppb has been reported in some locations around the world, such as Mauna Loa, on the island of Hawaii.^40^ The monitoring of air quality at an industrial estate at Uttarakhand, India (*Table 3*) revealed 15.0 μg/m^3^ and 23.0 μg/m^3^ of SO_2_ and NOx, respectively, categorizing the estate as being moderately to heavily polluted. The characterization of air quality in an industrial area in Incheon, South Korea revealed high levels of SO_2_ (17.0 μg/m^3^) and NOx (71.4 μg/m^3^). These levels of gaseous air pollutants have been attributed to vehicular emissions coupled with emissions from industries in the area.^41^

**Figure 6:**
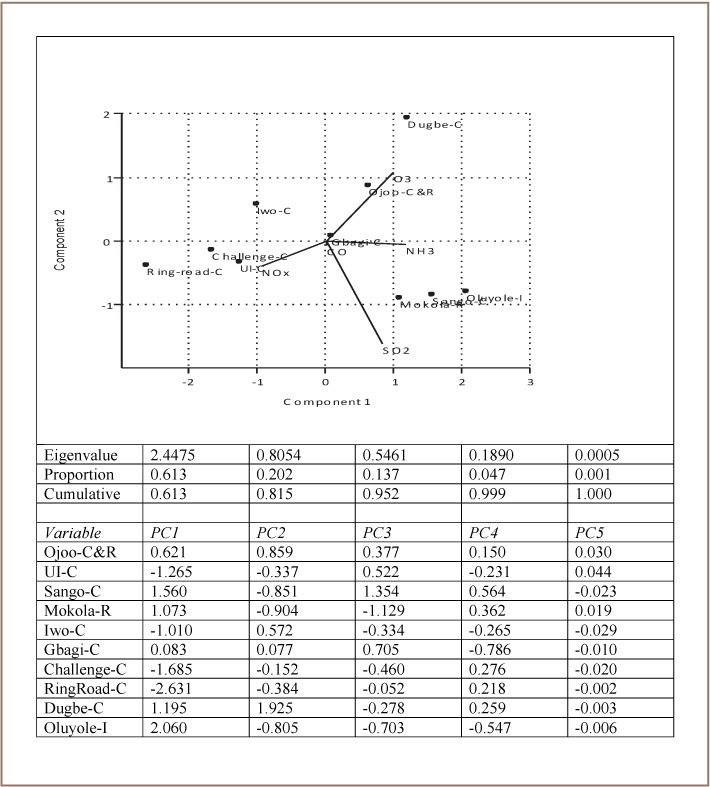
Biplot diagram illustrating the association of air pollutant levels with sampling location (C= commercial area, R= Residential area)

In this study, the first principal component in Figure 6 ranked the sampling locations from the least to most impacted by pollutant type. The Mokola, Sango and Oluyole sampling locations had similar heavy loadings for the first principal component of 61.3%. The sampling points cluster in the southeast quadrant where SO_2_ and NH_3_ appear as vectors. The Ring Road, together with the Challenge and UI sampling locations lie more towards the southwest where the vector is NOx. These sampling locations have similar heavy loadings for the second principal component of 20.2% (*Figure 6*). This implies that the biplot explains 81.5% of the variance of the axes for the relationship between the sampling locations and the concentrations of pollutant types. The eigenvalue quantifies how much variance is accounted for by each principal axis and the proportion can be expressed as a percentage. In addition, the Ojo sampling location lies towards the southeast with the Dugbe sampling location located further away. According to the diagram, NOx was predominant at the Ring road, Challenge, UI locations which are all commercial areas. SO_2_ and NH_3_ were emitted in large proportions at the Oluyole industrial area, Mokola and Sango, which are residential and commercial areas, respectively. The Ojoo sampling location, a commercial and residential area, was associated with a large proportion of ozone.

### Levels of Pollutants at Sampling Locations

High concentrations of NOx observed in all of the sampling locations compared to the control site were possibly due to heat emanating from motor vehicles on the highway and electric utilities from residential and industries that burn fuels. These sources are known to contribute nitrogen oxides when fuel is burned at high temperatures.^38^,^42^ Fossil fuel combustion contributes more than the natural sources. Vehicle exhaust is known to enhance the concentration of NOx in the atmosphere.^42^

Ground level ozone is not emitted directly into the air, but is formed by photochemical reactions of NOx and volatile compounds.^38^,^43^ Ozone gas appears to be pronounced at industrial locations due to photochemistry involving pollutants, which are released from various industrial and other anthropenic activities such as residential heating. Photochemical ozone production occurs by photo-oxidation of methane and CO in the presence of sufficient amounts of NOx.

The sources of NH_3_ are difficult to specify since the earth acts as a sink for indiscriminate excreta of humans by roadside and household coal consumption in residential areas, which are possible sources of ammonia emission in this study. Although ammonia is currently not included in national emission air quality standards, the presence of ammonia in a quantifiable measure highlights the urgency of the need for regular monitoring of NH_3_ emissions in the city. This monitoring has become imperative since the Nigerian Federal Ministry of Environment is particularly interested in a database on ammonia emissions from various categories of land usage. The agency noted that there are some environmental consequences associated with high atmospheric ammonia levels and its deposition. Soil acidification, aquatic eutrophication and odor emanation are a few of the environmental effects traceable to ammonia deposition.^44^

Substances used for fuel in transportation, in the heating of homes and in industrial processing could have been responsible for CO emission in the study areas. Another source of CO production in residential areas is cigarette burning. The CO emission source at the Oluyole industrial area is not attributed to vehicles only, but also an industrial plant. It is an indication that automobile exhaust is not the main contributor of CO in an urban atmosphere. Thus, industrial point source also contributed to CO levels at the Oluyole industrial estate, but its proportion could not be ascertained in this study.

### Variation of Pollutant Levels in Sampling Locations

In the daytime, the ensemble of nitric oxide (NO) and NO_2_ can undergo a sequence of reactions in the presence of global solar radiation.^45^ NO is a primary pollutant, while NO_2_ and O_3_ are secondary air pollutants formed via a set of reactions. In the morning, sunlight starts to induce a series of photochemical reactions during which NO is converted to NO_2_ and oxygen. During daytime hours, NO_2_ is photolyzed back to NO and oxygen atoms. The atom recombines with oxygen to regenerate O_3_. This cycling between NO and NO_2_ takes place within one minute during the day. The rapid exchange between NO and NO_2_ while ozone is regenerated might explain the poor correlation between NOx and ozone concentrations. In addition, NOx could possibly be oxidized in the daytime to nitric acid by a strong radical oxidant. Solar radiation and temperature affect the rate of chemical reaction that produces ozone. An increase in the values of these variables increases the rate of photolysis reaction. There was no rainfall during the sampling period as evidenced by the observed ambient temperature. The dry condition in the morning would have suppressed the production of ozone by reducing the rate of photolysis. This may be responsible for the low level of ozone in the morning.

The considerably high level of ammonia observed during morning rush hours is similar to the report that automobiles were found to be major contributors to the observed high level of ammonia in Houston, Texas, particularly in the morning.^46^ Automobiles are equipped with 3-way catalysts to control nitrogen oxide pollutants. The catalysts operate by constantly oscillating the air-to-fuel ratio in the engine and subsequently produce ammonia under reducing conditions.^47^ In the afternoon, other local emission sources such as burning, livestock production practices and industrial activities from different wind directions would have contributed to the elevated ammonia concentrations. In urban areas, industrial activities contribute to significant increases in local or regional ammonia levels.^48^

The variability in CO levels seems to depend on the location (industrial, residential or commercial) where CO measurements are taken. Carbon monoxide levels in most cities reach the daily maxima between 7:00 and 9:00 am coincidental with heavy morning rush hour automobile traffic.^39^ Daily CO concentrations were higher in the afternoon than in the morning possibly due to greater automobile traffic in commercial areas and higher industrial heating in the afternoon.

## Conclusions

The present study revealed that anthropogenic activities such as fossil fuel combustion, household coal consumption and indiscriminate excreta by humans around commercial, residential and industrial areas played a large role in increasing the background concentrations of SO_2_, NOx, O_3_, NH_3_ and CO. The increase was about 15, 31, 17, 5 and 3 times the background concentration for SO_2_, NOx, O_3_, NH_3_ and CO, respectively. Residential areas had the highest level of CO, followed by industrial and commercial areas. A reduction pattern in the levels of SO_2_, O_3_ and NH_3_ following the order of industrial > residential > commercial was obtained in this study for the sampling areas. The proportion of NOx for the residential area was similar to the proportion for the commercial area, but greater than the proportion for industrial areas. The level of SO_2_ exceeds the World Health Organization limits, indicating a need for long term air quality monitoring for a sustainable plan for air pollution management. The data provided in this study will assist in advising the general public according to the regulatory bodies of current air quality levels and pollution warnings.
